# Fluorometric Nanoscale Analysis of Bilirubin and Biliverdin in Human Cerebrospinal Fluid

**DOI:** 10.1021/acschemneuro.5c00265

**Published:** 2025-06-25

**Authors:** Paola Sist, Federica Tramer, Arianna Sartori, Paolo Manganotti, Sabina Passamonti

**Affiliations:** † Department of Life Sciences, 9315 University of Trieste, via Giorgieri 1, I-34127 Trieste, Italy; ‡ Neurology Unit, Department of Medical, Surgical and Health Sciences, 9315 University of Trieste, 34149 Trieste, Italy

**Keywords:** cerebrospinal fluid, bilirubin, biliverdin, fluorometric analysis

## Abstract

Cerebrospinal fluid (CSF) is a valuable source for the quantification of soluble, brain-specific biomarkers supporting the diagnosis of some neurological disorders, infectious diseases, and suspected subarachnoid hemorrhage. Given the increasing need to expand the basic knowledge of brain pathophysiology for disease biomarker discovery, we set the goal to quantify bilirubin and biliverdin in human CSF. Their concentrations are expected to reflect the level of brain heme catabolism, a key pathway involved in the biological response to oxidative stress. Here, we present the results of the CSF analysis by a specific and sensitive fluorometric method using the recombinant fusion protein HELP-UnaG (HUG). The concentrations of bilirubin and biliverdin in 50 human CSF samples were in the ranges of 14–340 and 0–66 nM, respectively. This assay can be easily implemented in small-scale laboratories and neurological units for the routine analysis of clinical CSF samples.

## Graphical abstract

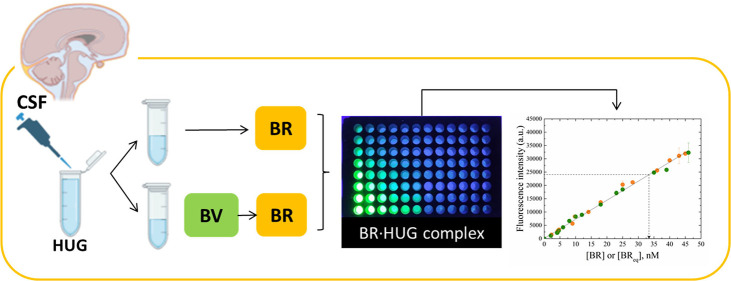

## Introduction

The cerebrospinal (CSF) is a biofluid in contact with the interstitial fluid of the brain and separated from the blood compartment by the blood-brain barrier. As a result, its chemical composition reflects brain metabolism and is markedly different from the plasma in terms of protein, ions, and brain-specific molecules or particles (i.e., extra-cellular vesicles). The unmatched value of CSF as a liquid biopsy is that organotypic biomarkers can be analyzed against a uniquely poor chemical interference background.^1^

The CSF is analyzed in various acute and chronic brain diseases, such as infections, traumatic injuries, hemorrhage, and neoplasms to obtain both a basic set of chemical parameters, such as the concentrations of protein, glucose, lactate, and cell counts,^2^ and other additional biomarkers to support the accurate diagnosis of other conditions, e.g., multiple sclerosis^3^ and Alzheimer's disease.^4^

Remnants of CSF samples are an invaluable resource for screening other low- and high-molecular weight components (metabolites, RNA, DNA) that may be either constitutively present in the CSF or appear because of brain or blood-brain barrier anomalies.^5^ This ancillary analysis of the CSF is expected to provide insights on essential pathophysiology aspects of the brain, needed for improving diagnostic accuracy and discernment, patient management and, importantly, biomarker discovery.^1^ Among the several chemical categories of emerging biomarkers, small molecules related to tryptophan, glutamine, or arachidonic acid pathways can be comprehensively screened by metabolomic analysis.^6^

The aim of this study was to quantify two poorly studied CSF metabolites, i.e., the bile pigments biliverdin and bilirubin that result from brain heme catabolism or, possibly, from their transfer across the blood-brain barrier.

The two possible sources of bile pigments in the CSF correspond to two categories of disease mechanisms and the related principles for diagnostic exploitation. The first disease mechanism concerns chronic neurodegenerative and neuroinflammatory diseases, in which oxidative stress is regarded as an early causal factor.^7^ Oxidative stress triggers upregulation of heme oxygenase 1 (HO-1), which catalyzes the oxygen- and NADPH-dependent cleavage of heme to release Fe^2+^ and the open tetrapyrrole molecule biliverdin (BV), which is then reduced to bilirubin (BR) by biliverdin reductase (BVR). In the mice brain, bilirubin acts as a powerful scavenger of superoxide anion, limiting the excitotoxicity linked to *N*-Methyl-d-aspartic acid (NMDA) receptor activation.^8^ The side product of HO enzymes is carbon monoxide (CO), which has been linked to several protective roles in the brain, by regulating the circadian clock or neuroinflammation.^9^ However, excessive upregulation of HO-1 is observed in several experimental models of neurodegenerative diseases, such as Alzheimer’s disease, Parkinson’s disease, and multiple sclerosis.^10^ For these reasons, it would be important to measure bilirubin in the CSF, to quantify the activation of heme catabolism. Nevertheless, there are only limited data on the presence of biliverdin and bilirubin in the CSF. Bilirubin is listed among the 468 compounds recorded in the CSF section of the human metabolome database (HMDB),^11^ with only one record of normal concentrations of 0–0.2 μM in adults of both sexes.^12^

The second disease mechanism concerns erythrocyte degradation and heme catabolism in subarachnoid hemorrhage, an acute event in which measurement of bilirubin in CSF may be needed if brain imaging is not adequate. With the abnormal presence of bilirubin, CSF takes on a yellow color named xanthochromia, which can be gauged by eye^13^ or direct spectrophotometry.^14^ The spectrophotometric method for quantifying bilirubin in CSF is not necessarily intuitive and has several limitations. Potential interfering substances, such as oxyhemoglobin and methemoglobin, can make accurate identification of bilirubin difficult. The absorption peak of oxyhemoglobin is at wavelengths between 410 and 418 nm, while that of bilirubin is between 450 and 460 nm, which represents a shoulder next to the oxyhemoglobin peak and requires accurate differentiation during analysis. In addition, the need for large volumes of CSF can be challenging.^15^

In this work we demonstrate the possibility to achieve quantification of both bilirubin and biliverdin in human nonhemorrhagic CSF samples in the nM range. We applied a 96-well plate fluorometric method based on high-affinity bilirubin binding to the HELP-UnaG fusion protein known as HUG,^16^ which features high precision and reproducibility^17^ and could be upgraded for the combined analysis of bilirubin and biliverdin.^18^ As in all UnaG-based assays,^19^ BR-dependent fluorescence is highly specific and free from hemoglobin interference. This analysis could be directly performed on CSF samples after centrifugation yielding results within the same day of the sample collection from patients.

## Results and Discussion

The prominent feature of the HUG-based analysis of BR and BV in biological samples under optimized conditions is that HUG affinity for BR is large enough to displace BR from sample binding sites and bind it in a reaction that goes to completion. Furthermore, for the same reason, several kinds of biological samples rich in bilirubin are usually diluted to keep the fluorescence of the HUG-BR complex within a measurable scale. Dilution brings down the concentration of interfering compounds of the matrix, ultimately sparing need of preanalytical sample preparation. In human plasma, where BR concentration is 6 μM, the matrix effect under the standard assay conditions was absent.^17^ To validate the HUG assay of bile pigments in CSF, a set of preliminary tests was performed using CSF without dilution or any pretreatment. Samples were obtained from adult human subjects visited at the onset of clinical signs of neurological disease and expected to have bile pigments not far from the normal concentration range.

### Method Verification

A spike-and-recovery test was performed, as recommended with never-tested sample types,^20^ by adding known amounts of BV and BR to known volumes of a single pool of CSF specimens to determine whether there was any interference from the CSF as a so far unknown analytical matrix. To experimentally obtain the expected BR and BV recoveries, identical spiking was performed on the solvent of the standard solution used for assay calibration, consisting of 4 g/L bovine serum albumin in phosphate saline buffered solution pH 8.5 (PBS-BSA). BR concentration was obtained as a direct measurement, while BV concentration was obtained as an indirect measurement, by applying a variant protocol where the HUG assay mixture is supplemented with biliverdin reductase (BVR) and its reaction cofactor NADPH to convert BV to BR.^18^ As shown in Table 1, no significant difference in BR or BV concentrations were found in CSF and the BSA solution, referred to as the observed and expected values, respectively, of all graded additions. These data show no matrix effect is acting on the assay.

**Table 1:** Analytical Recovery of Bile Pigments in the Human CSF^a^

	spike level	expected BSA	observed CSF	recovery %	*p*-value
BR	low	4.0 (±0.1)	3.9 (±0.6)	98	>0.998
	med	25.3 (±0.3)	26.4 (±1.2)	104	0.306
	high	43.5 (±0.7)	41.9 (±1.7)	96	0.078
BV	low	4.3 (±0.1)	4.2 (±0.3)	98	0.999
	med	23.1 (±0.3)	23.7 (±1.1)	103	0.818
	high	40.8 (±0.2)	41.4 (±2.3)	102	0.818

a Aliquots of CSF (observed) or PBS-BSA (expected) (20 μL; *n* = 4) were supplemented with small volumes (5 μL) of 0.16, 1.0, and 1.6 μM standard BR or BV solutions, to achieve low, medium and high increases of endogenous BR and BV levels. For the analysis within the linearity range of the standard HUG assay calibration (0–50 nM), both CSF and PBS-BSA were diluted 40-fold with 0.05 g/L HUG. Each aliquot was analyzed in quadruplicate. Data (means ± SD, *n* = 3) were obtained from the net fluorescence of spiked specimens, after subtraction of the basal fluorescence. Observed vs expected values were compared by t-Student test. Significant differences were set at *p* ≤ 0.05.

The range of linearity of the assay was characterized by using a single pool of CSF specimens serving as the solvent of standard BR and BV solutions having increasing concentrations. Figure 1 shows that the fluorescence signal was linearly related to BR and BV concentrations. There was a perfect equivalence of BR and BV, showing that the assay conditions (enzyme and cofactor) were optimal.^18^ Therefore, a single, common angular coefficient could be used (725 ± 26 A.U. nM^–1^) for fluorescence calibration. In turn, the latter was not statistically different (*p* = 0.203) from the standard reference angular coefficient obtained in PBS supplemented with 0.4 g/L BSA, which is 748 ± 42 A.U. nM^–1^.^17^ This finding demonstrates again the absence of the matrix effect on this assay.

**Figure 1:**
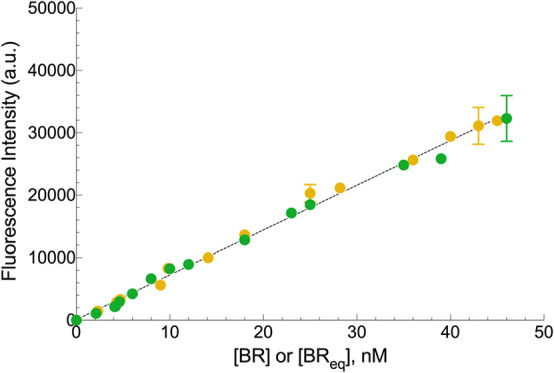
Calibration of BR-dependent fluorescence emission of HUG in CSF. BR (yellow circle) and BV (green circle) solutions in CSF were incubated with 0.05 g/L HUG at *T* = 25 °C and measured after 16 h. The CSF background fluorescence intensities of BR = 510 ± 27 and BV = 660 ± 26 were subtracted from data obtained at each BR or BV concentration. Data (means ± SD) were fitted by linear regression analysis (angular coefficient = 725 ± 26, R2 = 0.994, *n* = 6). BR_eq_ is BR derived from BV reduction.

### Stability of Bilirubin and Biliverdin in CSF

The stability of both bile pigments in the CSF upon storage at −80 °C for up to 4 months was tested on individual samples (*n* = 10 for BR and *n* = 8 for BV), randomly chosen from the set of available samples (*n* = 50). Data in Figure 2 show that each sample had its unique intrinsic BR and BV concentrations, which remained stable for 4 months in all cases. BV variability, though larger than that of BR, was of no statistical significance.

**Figure 2:**
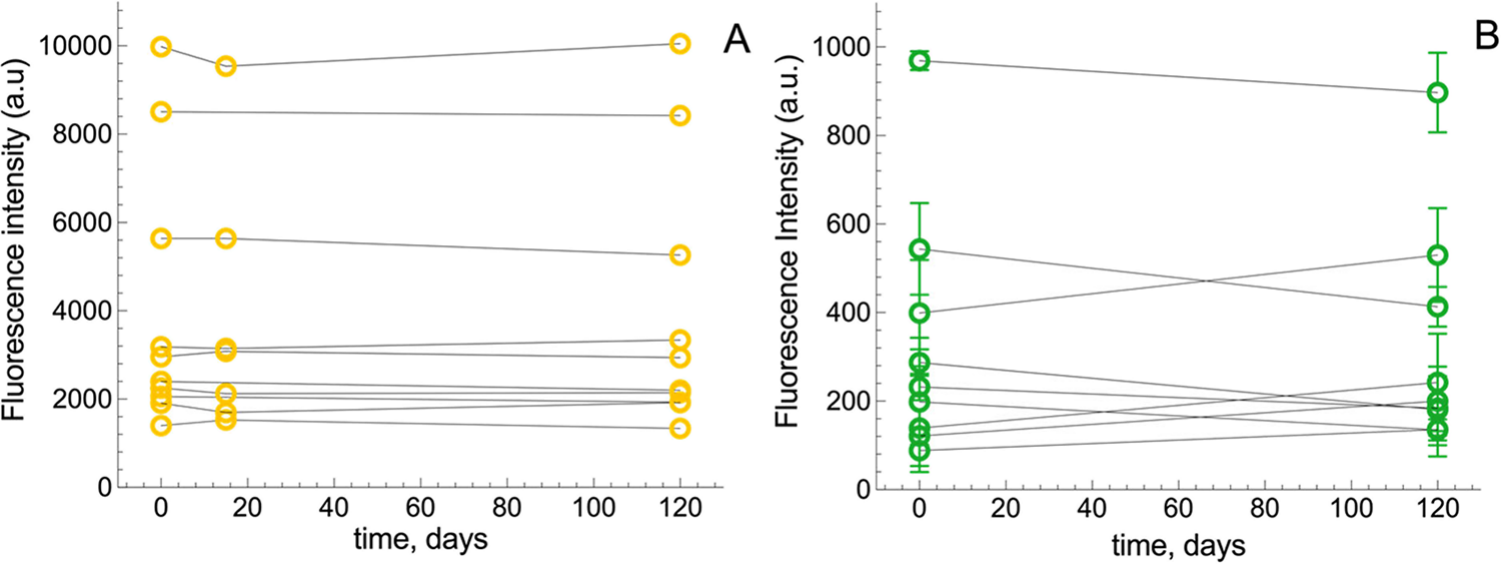
Stability of BR and BV in CSF upon storage. Samples of CSF with variable intrinsic BR and BV concentrations were divided in aliquots and stored at −80 °C. Aliquots were thawed at different times and analyzed for (A) BR (*n* = 10) and (B) BV (*n* = 9).

Like plasma or serum,^21^ BR in CSF also reacts to light and storage temperature. It decreases when exposed to light (2 h), while it remains stable at 4 °C for 3 weeks, as demonstrated by spectrophotometric measurements.^22^ The literature reports that the stability of several analytes, such as amyloid and tau proteins, in CSF is influenced by the time interval between collection and freezing rather than the final storage temperature.^23^ Storage of rapidly centrifuged and frozen CSF at −80 °C shows a two-year stability of amyloid proteins, while their concentration can be affected by repeated freeze–thaw cycles. This means that bile pigments can be analyzed in CSF samples archived in biobanks for the study of amyloidopathy. To our best knowledge, this the first evidence that bile pigments in the nM range are stable in frozen CSF samples, which will facilitate the exploitation of clinical biobanks.

### Bilirubin and Biliverdin Concentrations in CSF and Serum

The concentrations of BR and BV in both the CSF and serum obtained from 50 subjects (23 males and 27 females) are shown in Figure 3A. BR concentrations ranged from 14 nM up to 340 nM, with a median value of 30 nM. These data agree with that theorized by multiplying the serum bilirubin concentration by the albumin quotient^24^ or that in normal and normal concentrated CSF samples measured spectrophotometrically.^25,26^ Values in the same range have been found by applying the colorimetric diazo method^27^ or by using specific bilirubin antibodies.^28^

**Figure 3:**
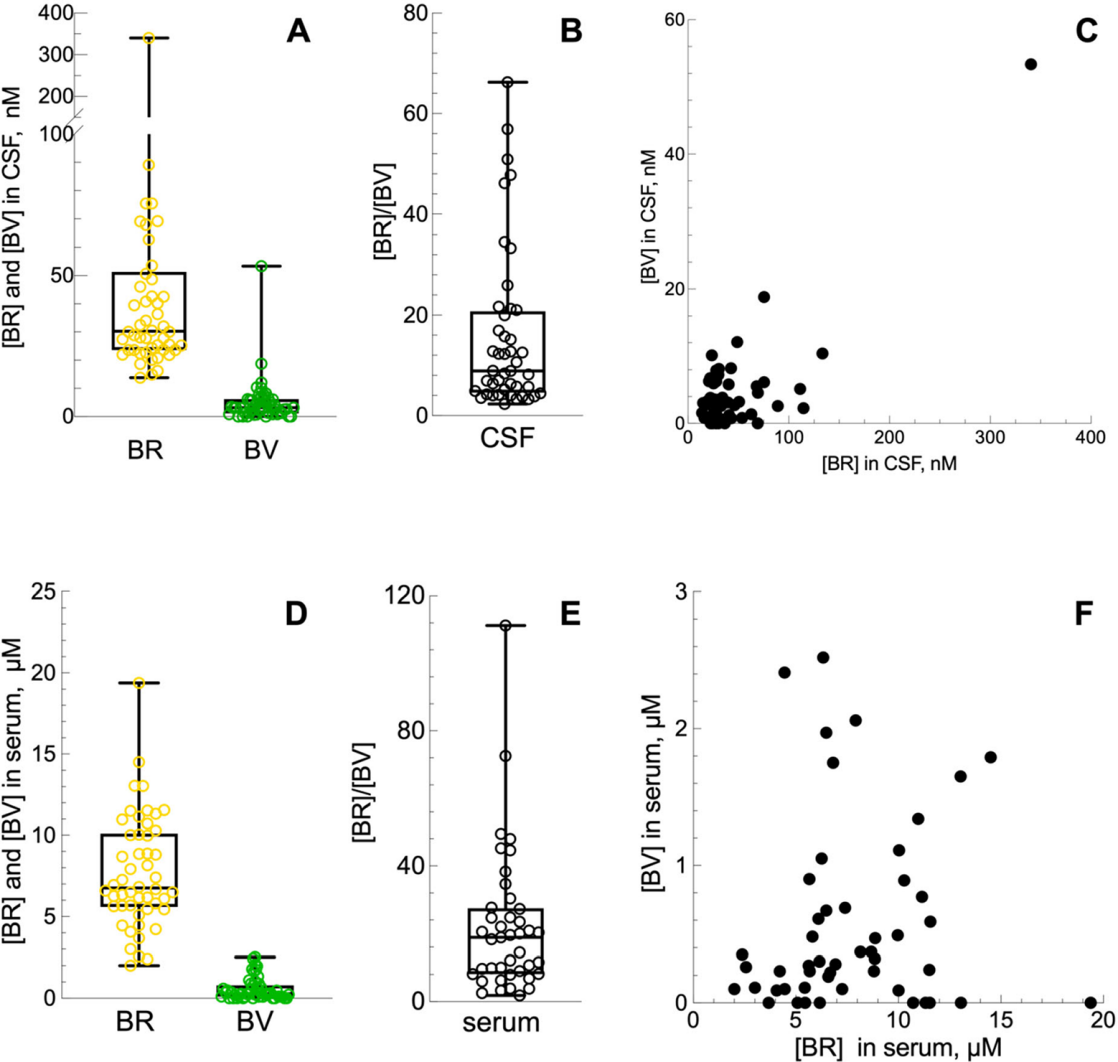
Concentrations of BR and BV in CSF and the ratio between the two pigments in all subjects. (A) BR (*n* = 50; mean = 47.37 nM; median = 30.32 nM; SD = 50.04; SEM = 7.08) and BV (*n* = 50; mean = 5.03 nM; median = 3.21 nM; SD = 7.88; SEM = 1.11). (B) BR/BV (*n* = 44; mean = 15.69; median = 8.91 nM; SD = 15.96; SEM = 2.41). (C) Correlation between BR and BV in CSF samples (*n* = 50). Data were analyzed using the Spearman’s correlation equation (rho = 0.159, *P* = 0.2673). (D) Concentrations of BR and BV in serum. BR (*n* = 50; mean = 7.71 μM; median = 6.75 μM; SD = 3.44; SEM = 0.49) and BV (*n* = 50; mean = 0.57 μM; median = 0.29 μM; SD = 0.68; SEM = 0.09). (E) BR/BV (*n* = 41; mean = 22.17; median = 18.87 μM; SD = 21.05; SEM = 3.29). (F) Correlation between BR and BV in serum samples (*n* = 50). Data were analyzed using the Spearman’s correlation equation (rho = 0.137, *P* = 0.3426).

BV ranged from undetectable levels up to 66 nM, with a median value of 3 nM. The BR/BV ratio (Figure 3B) ranged from 2.3. to 66.2, with a median value of 8.9. Since BV and BR are metabolically related, we looked if there is any correlation between their concentrations in each CSF sample. As shown in Figure 3C, the analysis revealed no significant correlation. This suggests that the levels of these two pigments in CSF are mutually independent.

In serum, BR concentrations ranged from 2.0 μM up to 19.38 μM, with a median value of 6.75 μM. BV ranged from undetectable levels up to a maximum of 2.52 μM, with a median value of 0.29 μM. The BR/BV ratio (Figure 4B) ranged from 1.85 to 111.2, with a median value of 18.87, which is double than in the CSF, but this difference was not statistically different.

**Figure 4:**
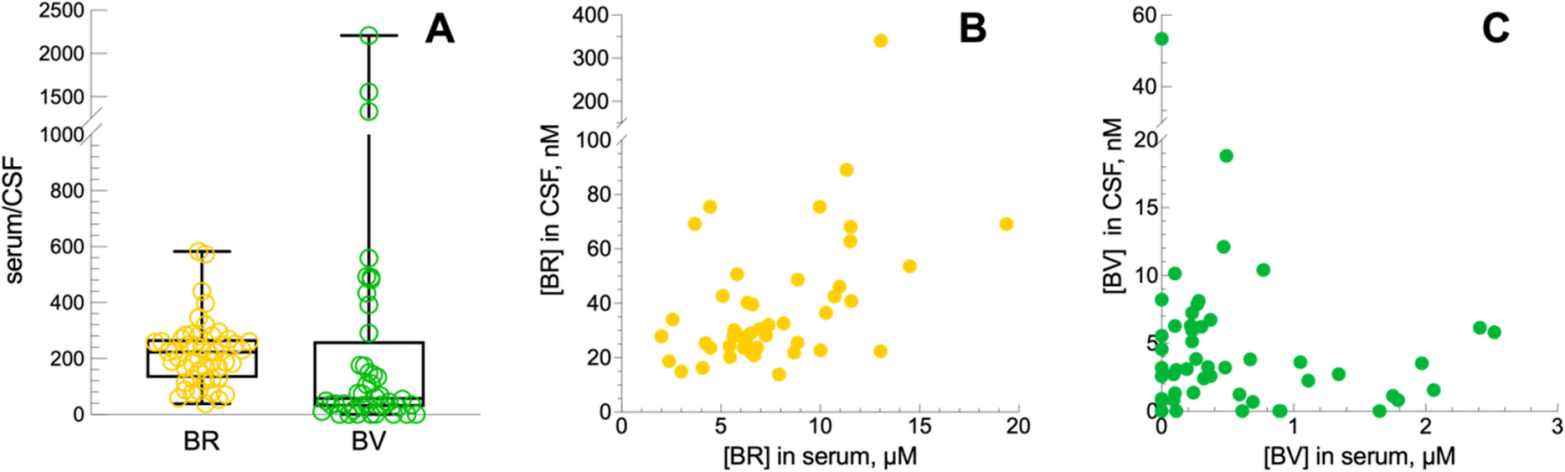
Partition ratio of BR and BV between serum and CSF. (A) BR serum/CSF ratio (*n* = 50; mean = 218.7; median = 222.9; SD = 114.9; SEM = 16.25) and BV serum/CSF ratio (*n* = 44; mean = 250.6; media = 57.97; SD = 457.8; SEM = 69.02). (B) Correlation between serum and CSF BR concentrations. Data correlation was analyzed by Spearman equation (rho = 0.462; *p* = 0.0007). (C) Correlation between serum and CSF BV concentrations. Data correlation was analyzed by Spearman equation (rho = −0.123; *p* = 0.395).

### Partitions of Bilirubin and Biliverdin between Serum and CSF

There is so far no established knowledge on the partition of bile pigments across the blood-brain barrier. The results of the analysis of the BR and BV serum/CSF partition ratio is shown in Figure 4A. The ratio ranged 38–582 for BR, with a median value of 223, whereas for BV the ratio ranged 0–2209, with a median value of 57.97. Both ratio values suggest tightness of the blood-brain barrier in most subjects. For the sake of comparison, the serum/CSF ratio of proteins available in a random subgroup of patients only (*n* = 32) was in the same range as BR (mean = 152.6; SD = 51.5; SEM = 27.0) and agrees with results published for albumin partition.^29,30^ Moreover, our data show that there is a good positive correlation between the BR concentrations in serum and CSF. Data reported in rat experiments^31^ and in early infancy subjects^32^ showed the same correlation. To date, it is not yet entirely clear whether, under physiological conditions, bilirubin crosses the blood-brain barrier as a solute or as a complex with albumin and follows therefore its transfer pathway through the blood-brain barrier.

Concerning BV, no significant correlation between serum and CSF values (Figure 4C) has been found. These data suggest that BV in the CSF originates from the brain metabolism and is not in equilibrium with the blood.

Overall, this is the first study that provides a set of values of BR and BV in the CSF that may be regarded as approximately normal in adults. There were no indications of blood-brain barrier disruption in the donors. Furthermore, it is one of the few studies that gives BR concentrations in the CSF in the absence of subarachnoid hemorrhage or other severely disrupting blood-brain barrier conditions.^33,34^ These data will enrich the records in the CSF metabolome database. Since CSF is a biological fluid difficult to obtain, especially from healthy volunteers for the study of heme metabolism and oxidative stress, the HUG assay can support research using preclinical experimental models in neuroscience.

### Advantages and Limitations of the Method

The HUG assay for the analysis of BV and BR has several advantages, such as: (1) Elevated specificity and sensitivity,^17^ which is suitable for the direct analysis of CSF, with no preanalytical sample preparation. This results in limiting the measurement precision uncertainties. (2) User-friendly implementation in small-scale laboratories and in neurology units equipped with basic instrumentation. (3) Availability of detailed protocols to produce HUG,^35^ prepare BR standards,^36^ and implement the assay.^17,18^ (4) Low cost. Therefore, its application for the screening of CSF remnants is expected to promote advances in the field of basic and applied neurological studies.

The limitations of the assay that needs to be addressed are that neither HUG nor an HUG assay kit are commercially available. At present, HUG must be produced in house or in partnership with public or private production units. A HUG assay kit, providing microwell plates and the basic reagents would overcome this limitation. Another limitation is that the HUG assay is not yet ready to be automated and implementable in the routine clinical diagnostics. More data on the value of bile pigments as diagnostic or prognostic biomarkers are needed.

## Materials and Methods

### Reagents

Analytical grade chemicals purchased from Merck were Bilirubin (BR, purity 99%), Bovine Serum Albumin fraction V (BSA, purity > 98%), Biliverdin (BV), Biliverdin reductase A human (BVR, 1500 IU/mL), Dulbecco’s Phosphate Buffered Saline (PBS), NADPH tetrasodium salt reduced form, Dimethyl sulfoxide (DMSO), Sodium hydroxide (NaOH). Ultrapure water Milli-Q was used to prepare each solution. HUG was synthesized and purified as described in detail.^35^ Black, 96-well microplates (Nunc, purchased by Thermofisher, code 237107; polystyrene, sterile, non-treated surface).

### Patients and Samples

A group of 50 subjects (Table 2) were visited at the Clinical Unit of Neurology, Department of Medicine, Surgery, and Health Sciences, University of Trieste, Trieste, Italy, because presenting early symptoms of neurological disease and required the standard analysis of CSF for diagnosis. The CSF sampling protocol was implemented at 8.30–10.30 a.m. CSF (0.5–1 mL) was collected by lumbar puncture in polypropylene tubes. The protocol included collection of blood by venipuncture. Both CSF and blood samples were centrifuged (2000 *g* for 10 min at 4 °C) and their supernatants were stored at −80 °C.

**Table 2:** Characteristics of Study Subjects

parameters		total	male (*n* = 23)	female (*n* = 27)
age (years)	average ± SD	58.8 ± 16.6	62.6 ± 13.5	55.5 ± 18.4
	range (min–max)	(21–84)	(36–84)	(21–79)
BMI	average ± SD	24.2 ± 3.3	24.4 ± 2.5	24.1 ± 4.0
	range (min–max)	(17.3–33.1)	(20.1–29.4)	(17.3–33.1)

This study was approved as a monocentric observational retrospective study by the Ethics Committee of the University of Trieste and conducted in accordance with the Declaration of Helsinki.

### Analysis of Bilirubin and Biliverdin in CSF and Serum

Within 1 week, remnants of both CSF and serum samples were thawed and analyzed by the HUG fluorometric assay for quantification of bilirubin (BR)^17^ and biliverdin (BV).^18^ Aliquots of CSF (160 μL) or serum (8 μL) were added to HUG solution (0.05 mg/mL in the assay medium) to obtain a final volume of 1.6 mL. The diluted samples (both CSF and serum) were then divided into two 0.8 mL-aliquots, one for bilirubin and the other for biliverdin quantification (Scheme 1). For analysis of BR, 200 μL was added directly to the multiwell plate in four replicates, whereas for BV, 5 μL of enzymatic solution was added to the second 800 μL aliquot (BVR final concentration 0.1875 mU/μL, NADPH 0.1 mM) and then added to the multiwell plate in four replicates. BR and BV standard solutions (5, 10, 25, 50 nM), prepared according to the protocol,^36^ were included in the 96-well plate for fluorescence calibration. The microtiter plate was incubated at *T* = 25 °C, and fluorescence intensity (λ_ex_ = 485 nm, λ_em_ = 528 nm; *T* = 25 °C) was recorded after 16 h in a benchtop multiplate reader (Synergy H1; BioTek, Winooski, VT). A single value of intrinsic fluorescence was obtained by adding CSF or serum (20 or 1 μL, respectively) to a final volume of 0.2 mL assay medium, and used to correct sample fluorescence. Bilirubin concentration in the wells supplemented with BVR and NADPH was regarded as the sum BR + BV = Total BR (TBR). BV was obtained by subtraction of the BR value (recorded in the wells without BVR and NADPH) from TBR. After fluorescence recording, BR or BV concentrations were calculated using the angular coefficient of the calibration curve.

**Scheme 1:**
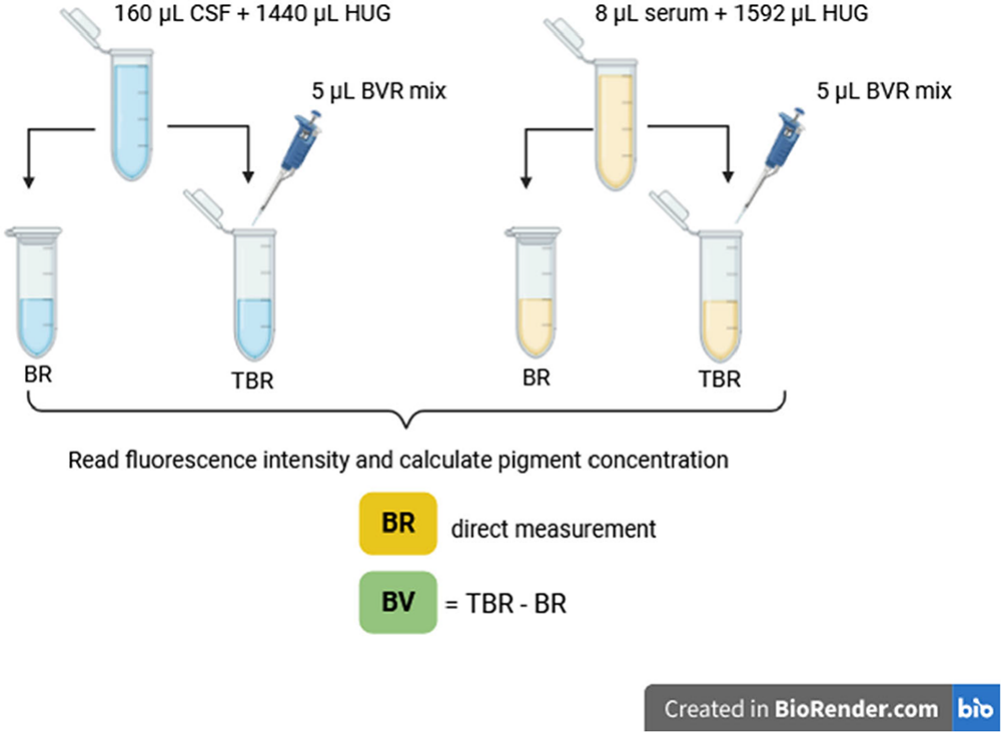
Sample Preparation for BR and BV Quantification

### Statistical Analyses

All data were analyzed and plotted using GraphPad Prism 10.1.0 (GraphPad Software). Unpaired Student *t* test was performed using standard significance level (α = 0.05).

## Abbreviations

BR: bilirubin
BV: biliverdin
CSF: cerebrospinal fluid
HUG: HELP-UnaG
HELP: human elastin-like polypeptide
